# Associations between ethylene oxide exposure and biological age acceleration: evidence from NHANES 2013–2016

**DOI:** 10.3389/fpubh.2024.1488558

**Published:** 2024-11-27

**Authors:** Xinyun Chen, Fangyu Shi, Wenhui Yu, Chunying He, Shenju Gou, Ping Fu

**Affiliations:** ^1^Department of Health Management, Health Management Center, General Practice Center, West China Hospital, Sichuan University, Chengdu, China; ^2^Department of Nephrology, Institute of Kidney Diseases, West China Hospital, Sichuan University, Chengdu, China

**Keywords:** ethylene oxide, biological aging, phenotypic age, KDM-AA, NHANES

## Abstract

**Introduction:**

Population aging is a global concern, with the World Health Organization predicting that by 2030, one in six individuals worldwide will be 60 years or older. Ethylene oxide (EO) is a widely used industrial chemical with potential health risks, including associations with age-related diseases. This study investigates the relationship between EO exposure and biological age acceleration.

**Method:**

Data from the National Health and Nutrition Examination Survey (NHANES) 2013–2016 were analyzed, including 3,155 participants after exclusions. Blood EO levels were measured using hemoglobin adducts (HbEO). Biological age acceleration was assessed using two methods: Phenotypic Age Acceleration (PhenoAgeAccel) and Klemera-Doubal Method Age Acceleration (KDM-AA). Linear and logistic regression models were applied, adjusting for various covariates, and restricted cubic spline (RCS) regression was used to explore non-linear associations.

**Results:**

Higher EO exposure was significantly associated with increased PhenoAgeAccel and KDM-AA across all models. In the continuous model, substantial positive associations were observed (PhenoAgeAccel: *β* = 0.73, *p* < 0.001; KDM-AA: *β* = 0.66, *p* < 0.001) in Model 3. Quintile analysis indicated a trend of increasing biological age acceleration with higher EO exposure. RCS regression demonstrated a significant linear relationship between EO exposure and PhenoAgeAccel (*p* for non-linearity = 0.067), as well as with KDM-AA (*p* for non-linearity = 0.083). Subgroup and interaction analyses revealed significant modifying effects by factors such as body mass index, gender, diabetes status, and physical activity level.

**Conclusion:**

Our study demonstrates a significant association between EO exposure and accelerated biological aging. These findings highlight the need for further prospective and mechanistic studies to validate and explore this phenomenon.

## 1 Introduction

Population aging is a global challenge, with the World Health Organization predicting that by 2030, approximately one in six individuals worldwide will be 60 years or older.^1^ As the population ages, the prevalence of age-related conditions such as cardiovascular disease, diabetes, and cancer is expected to rise, placing increasing pressure on healthcare systems and society (1). Aging is a complex process marked by progressive changes in tissue structure and physiological function, yet the rate and nature of these changes can differ substantially across individuals. Chronological age (CA), which simply measures the number of years a person has lived, does not fully capture the biological processes driving aging (2). In contrast, biological age (BA), also referred to as physiological age, serves as a more accurate indicator of an individual’s true aging status and associated health risks (3). BA reflects the cumulative impact of genetic, lifestyle, and environmental factors on the body, providing insights into the functional state of organs and systems. This makes it a more refined measure of health status and a better predictor of susceptibility to age-related conditions compared to *CA.* Assessing BA can therefore inform personalized health interventions, track the effectiveness of lifestyle modifications or treatments, and improve predictions of life expectancy and quality of life. Given the expected rise in age-related diseases as the global population continues to age (4, 5), understanding the factors that influence BA and identifying modifiable elements that can decelerate its progression are crucial for reducing the impact of aging on public health, increasing longevity, and enhancing quality of life.

The relationship between ethylene oxide (EO) exposure and BA has gained increasing attention in environmental health research due to the potential health risks associated with this highly reactive compound. EO is a versatile and widely utilized chemical in various industrial processes, including the production of ethoxylated compounds, ethanolamines, and ethylene glycol ethers (6). It is also commonly used in industries such as cleaning, pharmaceuticals, and printing and dyeing. Additionally, EO serves as an effective bactericidal agent, frequently applied in medical disinfection and industrial sterilization (7). EO exists as a gas at room temperature, making inhalation the primary route of exposure for humans. The general population is mainly exposed to EO through contaminated air, cigarette smoke, and vehicle exhaust (8). Once inhaled, EO rapidly enters the bloodstream, where it is distributed throughout the body (9, 10). As a highly reactive compound, EO readily interacts with nucleic acids and proteins, leading to the formation of hemoglobin adducts of ethylene oxide (HbEO), which are considered reliable biomarkers for assessing EO exposure (11, 12).

The reactivity of EO also induces the formation of reactive oxygen species (ROS), contributing to oxidative stress and potentially accelerating biological aging processes (13). Its interactions with nucleic acids can form DNA adducts and activate DNA repair mechanisms, which may lead to telomere shortening and other markers of accelerated aging (14). Furthermore, EO functions as an alkylating agent, inducing gene mutations and chromosomal aberrations by forming adducts with DNA, thereby increasing the risk of genotoxic effects (15, 16). Prolonged exposure to EO, particularly in industrial settings, has been linked to an elevated risk of hematopoietic system tumors and breast cancer (17, 18). Additionally, studies suggest potential associations between EO exposure and age-related diseases, such as cardiovascular diseases (CVD), chronic obstructive pulmonary disease (COPD), and diabetes mellitus (DM) (8, 19, 20). Despite these findings, research has not yet directly examined the correlation between EO exposure and the biological aging process, highlighting a critical gap in understanding the long-term health impacts of EO.

Given the increasing worries about the effects of environmental pollution on public health, this study sought to investigate the potential link between EO exposure and the acceleration of BA utilizing data of the National Health and Nutrition Examination Survey (NHANES), a comprehensive survey conducted in the United States, from 2013 to 2016. The research aimed to provide novel insights into the environmental risk factors contributing to aging.

## 2 Materials and methods

### 2.1 Study population

**Figure 1:**
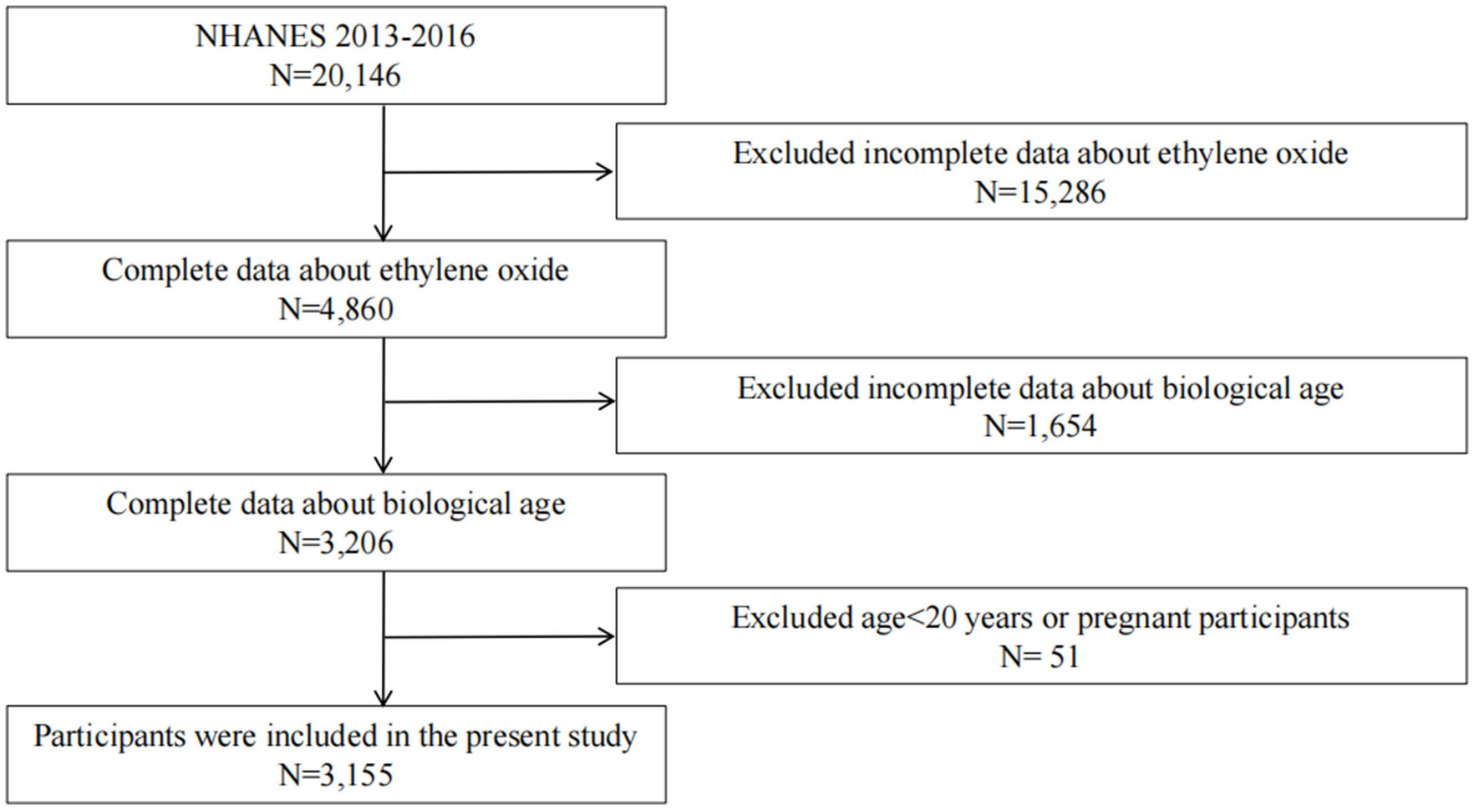
Flowchart depicting the selection process of study participants.

The NHANES is an extensive series of cross-sectional studies that focus on the population of the United States.^2^ Data are gathered via in-person interviews, physical and physiological evaluations, and detailed laboratory tests. 20,146 participants were enrolled from 2013 to 2016. However, 15,286 participants were excluded due to missing data on EO. Furthermore, 1,654 participants were excluded because of incomplete data on biological age (Phenotypic age (PhenoAge) and Klemera–Doubal Method biological age (KDM-BA)). After also excluding pregnant women and individuals younger than 20 years, the final sample included 3,155 individuals (Figure 1).

### 2.2 Data collection

#### 2.2.1 Blood ethylene oxide levels

HbEO is a highly sensitive method for detecting ethylene oxide (EO) exposure by quantifying hemoglobin adducts. In this study, morning blood samples were collected from participants who had fasted for at least 9 h at Mobile Examination Centers (MECs). The samples were drawn into ethylenediaminetetraacetic acid (EDTA) tubes and transported to the laboratory for processing. Upon arrival, red blood cells were thoroughly washed with isotonic saline to remove plasma and buffy coat, leaving only packed red blood cells. These cells were then stored at −30°C to preserve their integrity and prevent hemoglobin adduct degradation.

The detection process involved derivatizing the N-terminal valine of hemoglobin protein chains that had reacted with EO, forming N-[2-carbamoyl ethyl] valine and N-[2-hydroxycarbamoyl-ethyl] valine adducts. This derivatization was achieved using a modified Edman reaction, which specifically targeted valine adducts. The reagents reacted with the hemoglobin adducts to form stable derivatives, which were then separated and quantified using High-Performance Liquid Chromatography coupled with Tandem Mass Spectrometry (HPLC-MS/MS). The HPLC-MS/MS system provided high-precision separation of derivatives through liquid chromatography, followed by mass spectrometric detection to identify and quantify the specific adducts based on their mass-to-charge ratio. The system was calibrated using EO standards to ensure accurate quantification.

A commercial assay kit from Tech Diagnostics (Anaheim, CA) was used for derivatization and detection, providing all necessary reagents and standardized instructions. For further methodological details, including calibration curves, sensitivity, and reproducibility, please refer to: https://wwwn.cdc.gov/Nchs/Nhanes/2015-2016/ETHOX_I.htm.

#### 2.2.2 Outcome variable

The BioAge R package offers a suite of algorithms designed for calculating biological aging using NHANES data (21).^3^ This study utilized two specific methods: PhenoAge and KDM-BA. PhenoAge is derived from an algorithm that assesses mortality risk based on a range of biomarkers, providing a comprehensive evaluation of an individual’s aging pace and susceptibility to age-related diseases (22). KDM-BA employs regression analysis of biomarkers against chronological age to understand health status and the risk of age-related conditions (23). In this study, various biological markers, including systolic blood pressure, blood creatinine, urea nitrogen, albumin, total cholesterol, glycated hemoglobin, lymphocyte percentage, mean erythrocyte volume, leukocyte count, and alkaline phosphatase, were used to assess the aging phenotype.

To assess the rate of aging, this study employed two indicators of accelerated biological aging: Phenotypic Age Acceleration (PhenoAgeAccel) and Klemera-Doubal Method Age Acceleration (KDM-AA). PhenoAgeAccel is calculated as the residual from regressing PhenoAge on chronological age, where a positive residual reflects accelerated aging and a negative residual indicates decelerated aging. To enhance the robustness of the findings, KDM-AA was included as an additional metric. Similar to PhenoAgeAccel, KDM-AA is derived from the residual of regressing KDM-BA on chronological age. By incorporating KDM-AA, which utilizes a distinct methodological approach to estimate biological age, this study provides an extra layer of validation, ensuring that the observed associations with accelerated aging are not reliant on a single aging metric.

#### 2.2.3 Assessment of covariates

Covariate data were collected through a combination of surveys, physical examinations, and laboratory tests. The surveys gathered information on participants’ age, sex, ethnicity, smoking status, alcohol consumption, caloric intake, and health conditions, including DM, hypertension, CVD, and cancer. Poverty income ratio (PIR) is a ratio of family income to poverty threshold. Physical assessments involved measuring parameters such as blood pressure. Education levels were grouped into below high school, high school graduates, and above high school education. CVD was determined in participants with a history of congestive heart failure, coronary artery disease, angina, or myocardial infarction (24). Hypertension was defined as having a systolic pressure of ≥130 mmHg, diastolic pressure of ≥90 mmHg, a medical diagnosis of hypertension, or the use of antihypertensive medications (25). DM was diagnosed based on a previous diagnosis, use of diabetes medication or insulin, fasting blood glucose levels of ≥126 mg/dL, glucose levels of ≥200 mg/dL after 2 h oral glucose intake, or HbA1c levels of ≥6.5% (26).

Alcohol consumption was classified into three groups: never (fewer than 12 drinks in a lifetime), low-to-moderate (up to one drink per day for women and up to two drinks per day for men in the past year), and high (more than one drink per day for women and more than two drinks per day for men in the past year) (27). Smoking status was categorized as never (fewer than 100 cigarettes in a lifetime), former (over 100 cigarettes in a lifetime but not currently smoking), and current smokers (over 100 cigarettes in a lifetime and currently smoking) (28). Physical activity levels were quantified by assigning a metabolic equivalent (MET) value to each activity listed in the questionnaire, based on the compendium of activity energy costs (29).

### 2.3 Statistical analyses

Continuous variables were presented as adjusted means ± standard deviations or medians with interquartile ranges, while categorical variables were represented using unweighted counts and weighted percentages. Group differences were evaluated using χ2 tests and one-way ANOVA. To address missing values in various covariates, multivariate imputation by chained equations was applied, using random forest methods to complete the dataset (30). Weighted multiple linear regression was employed to estimate regression coefficients (*β*) and 95% confidence intervals (95% CI) for EO exposure in relation to biological age acceleration. Both PhenoAgeAccel and KDM-AA were used as outcome variables in these analyses, with KDM-AA specifically incorporated as a robustness check to ensure the consistency of the results. Weighted logistic regression models were used with PhenoAgeAccel and KDM-AA values greater than zero as the outcome variables. Model 1 included no confounders. Model 2 adjusted for age, sex, and ethnicity. Model 3 included additional adjustments for lifestyle factors such as alcohol consumption (31), smoking status (32), education level (33), body mass index (BMI) (34), physical activity (35), and medical history [CVD (36), hypertension (37), DM (38), and cancer (39)]. Restricted Cubic Spline (RCS) regression with four knots was used to examine potential non-linear associations between EO exposure and PhenoAgeAccel (40). Subgroup and interaction analyses were conducted to explore the interactive effects of various covariates identified as potential effect modifiers (40). All statistical analyses were carried out using R software, version 4.2.2 (R Foundation, Austria).^4^ A *p*-value of less than 0.05 was considered statistically significant.

## 3 Results

### 3.1 Participant characteristics

The baseline characteristics of 3,155 participants were divided into three tertiles based on PhenoAgeAccel, with an average age of 47.63 years and 50.5% male (Table 1). Participants in Tertile 3 tended to be older, more likely to be male, have a lower poverty income ratio, be less educated, have a higher BMI, smoke more, have lower levels of physical activity, and have higher rates of CVD, hypertension, and DM. Laboratory measures indicate that participants in Tertile 3 are prone to elevated serum creatinine levels and lower albumin levels. Additionally, EO levels progressively rise across the tertiles, highlighting a potential association between higher phenotypic aging and increased EO exposure.

**Table 1:** Baseline characteristics of participants, weighted.

	Total *n* = 3,155	Phenotypic age acceleration			
		Tertiles 1 *n* = 1,052	Tertiles 2 *n* = 1,051	Tertiles 3 *n* = 1,052	*p-*value
Age, years	47.63 ± 16.96	48.80 ± 16.33	45.55 ± 17.17	48.59 ± 17.21	0.013
Male, *n* (%)	1,576 (50.5)	357 (35.3)	549 (53.5)	670 (64.2)	<0.001
Race*, n* (%)					<0.001
Mexican American	483 (9.1)	172 (9.5)	171 (9.7)	140 (7.9)	
Non-Hispanic White	1,251 (66.2)	371 (65.2)	435 (66.9)	445 (66.5)	
Non-Hispanic Black	618 (10.5)	160 (7.8)	200 (10.1)	258 (13.8)	
Others	803 (14.2)	349 (17.5)	245 (13.3)	209 (11.7)	
Poverty income ratio	2.96 (1.43, 5.00)	3.25 (1.59, 5.00)	3.11 (1.45, 5.00)	2.58 (1.26, 4.48)	0.002
Education, *n* (%)					<0.001
Less than high school	318 (5.5)	115 (6.1)	99 (5.2)	104 (5.2)	
High school	1,077 (30.1)	302 (23.8)	348 (28.7)	427 (38.4)	
More than high school	1,760 (64.4)	635 (70.1)	604 (66.0)	521 (56.4)	
Cardiovascular disease, *n* (%)	312 (8.0)	62 (5.3)	79 (6.5)	171 (12.8)	<0.001
Hypertension, *n* (%)	1,125 (31.4)	272 (25.0)	342 (27.3)	511 (43.0)	<0.001
Diabetes, *n* (%)	570 (15.0)	95 (8.4)	130 (9.2)	345 (28.7)	<0.001
Cancer, *n* (%)	300 (10.6)	85 (10.3)	105 (11.1)	110 (10.5)	0.877
Diet energy, kcal/day	1974 (1,520, 2,467)	1906 (1,467, 2,356)	1992 (1,549, 2,530)	2043 (1,549, 2,543)	0.003
Body mass index, kg/m^2^	29.21 ± 6.83	27.25 ± 5.64	29.23 ± 6.51	31.38 ± 7.66	<0.001
Smoking, *n* (%)					<0.001
No	1,778 (55.9)	683 (61.8)	622 (60.1)	473 (44.7)	
Former	748 (25.6)	243 (27.0)	245 (23.7)	260 (26.0)	
Current	629 (18.6)	126 (11.3)	184 (16.3)	319 (29.2)	
Drinking, *n* (%)					0.235
No	1,000 (23.8)	396 (27.0)	306 (21.2)	298 (23.2)	
Former	1,936 (68.0)	601 (65.7)	660 (69.3)	675 (69.1)	
Current	219 (8.2)	55 (7.4)	85 (9.5)	79 (7.8)	
MET, *n* (%)					0.021
<600 min/week	1,251 (35.5)	404 (34.7)	393 (32.2)	454 (40.1)	
600–3,999 min/week	1,122 (37.2)	423 (41.0)	366 (37.4)	333 (32.9)	
≥4,000 min/week	782 (27.2)	225 (24.3)	292 (30.4)	265 (27.0)	
WBC, 1000 cells/μL	7.35 ± 2.14	6.21 ± 1.48	7.21 ± 1.74	8.78 ± 2.34	<0.001
Albumin, g/L	43.38 ± 3.13	44.20 ± 2.86	43.52 ± 2.97	42.33 ± 3.29	<0.001
Total cholesterol, mmol/L	4.91 ± 1.04	5.05 ± 0.98	4.93 ± 1.03	4.72 ± 1.07	0.001
Alkaline phosphatase, U/L	62.00 (51.00, 76.00)	59.00 (49.00, 71.00)	62.00 (51.00, 75.00)	67.00 (54.00, 82.00)	<0.001
Serum creatinine, μmol/L	75.14 (63.65, 88.40)	66.30 (57.46, 76.02)	77.79 (66.30, 87.52)	86.63 (73.37, 101.66)	<0.001
Klotho, pg/mL	782.50 (641.00, 973.09)	800.12 (652.56, 977.00)	792.16 (644.86, 981.98)	757.51 (613.52, 957.36)	0.005
Ethylene oxide (pmol/g Hb)	19.58 (14.12, 36.63)	18.02 (13.13, 25.31)	19.39 (14.18, 32.91)	23.22 (15.36, 108.85)	<0.001

n (%), Unweighted numbers (weighted percentage); MET, Metabolic Equivalent of Task; WBC, White Blood Cell.

### 3.2 Relationship between ethylene oxide exposure and biological age acceleration

Table 2 presents the linear regression analysis examining the associations between EO exposure and PhenoAgeAccel. In the continuous model, a significant positive association is observed across all models (Model 1: *β* = 1.05, Model 2: *β* = 0.97, Model 3: *β* = 0.73, all *p*-values <0.001). When categorized into quintiles, the analysis reveals a trend of increasing PhenoAgeAccel with higher EO exposure. Participants in Quintile 4 have a significantly higher PhenoAgeAccel compared to those in Quintile 1 across all models (Model 1: *β* = 2.66, Model 2: *β* = 2.43, Model 3: *β* = 1.42, all *p*-values <0.001). The trend test confirms the robustness of this association (*p* for trend <0.001 in all models). Table 3 presents the results of logistic regression analysis. Higher EO exposure is linked to increased odds of elevated PhenoAgeAccel across all three models (Model 1: OR = 1.54, Model 2: OR = 1.53, Model 3: OR = 1.42, all *p*-values <0.001). When categorized into quintiles, Quintile 4 shows significantly higher odds compared to Quintile 1 across all models (Model 1: OR = 3.03, Model 2: OR = 2.94, Model 3: OR = 2.02, all *p*-values <0.01).

**Table 2:** Linear regression analysis for the associations between ethylene oxide exposure and Phenotypic Age Acceleration, weighted.

	Phenotypic age acceleration					
	Model 1		Model 2		Model 3	
	β (95%CI)	*p-*value	β (95%CI)	*p-*value	β (95%CI)	*p-*value
Continuous^†^	1.05 (0.90, 1.20)	<0.001	0.97 (0.83, 1.12)	<0.001	0.73 (0.52, 0.94)	<0.001
Categories						
Quintiles 1	Reference		Reference		Reference	
Quintiles 2	0.42 (0.01, 0.84)	0.046	0.40 (−0.01, 0.80)	0.052	0.27 (−0.11, 0.65)	0.166
Quintiles 3	0.75 (0.31,1.20)	<0.001	0.70 (0.27, 1.13)	0.001	0.44 (0.03, 0.85)	0.035
Quintiles 4	2.66 (2.24, 3.10)	<0.001	2.43 (2.01, 2.86)	<0.001	1.42 (0.84, 2.00)	<0.001
*p* for trend		<0.001		<0.001		<0.001

Model 1 was unadjusted. Model 2 was adjusted for age, sex, and race. Model 3 was further adjusted for the variables in Model 2, along with education level, alcohol consumption, smoking status, physical activity, cardiovascular disease (CVD), hypertension, diabetes, cancer, occupation, and poverty income ratio (PIR). Continuous^†^, ln-transformed concentration of ethylene oxide. p for trend was tested by incorporating the variables of the median of each quartile into the regression model.

**Table 3:** Logistic regression analysis for the associations between ethylene oxide exposure and the risk of Phenotypic Age Acceleration, weighted.

	Phenotypic age acceleration					
	Model 1		Model 2		Model 3	
	OR (95%CI)	*p-*value	OR (95%CI)	*p-*value	OR (95%CI)	*p-*value
Continuous^†^	1.54 (1.41, 1.69)	<0.001	1.53 (1.39, 1.69)	<0.001	1.42 (1.19, 1.70)	0.002
Categories						
Quintiles 1	Reference		Reference		Reference	
Quintiles 2	1.24 (0.85, 1.80)	0.254	1.22 (0.82, 1.80)	0.305	1.16 (0.71, 1.88)	0.490
Quintiles 3	1.35 (0.95, 1.91)	0.091	1.30 (0.92, 1.83)	0.125	1.16 (0.88, 1.72)	0.386
Quintiles 4	3.03 (2.33, 3.93)	<0.001	2.94 (2.22, 3.89)	<0.001	2.02 (1.30, 3.13)	0.008
*p* for trend		<0.001		<0.001		0.011

OR: weighted odds ratio. Model 1 was unadjusted. Model 2 was adjusted for age, sex, and race. Model 3 was further adjusted for the variables in Model 2, along with education level, alcohol consumption, smoking status, physical activity, cardiovascular disease (CVD), hypertension, diabetes, cancer, occupation, and poverty income ratio (PIR). p for trend was tested by incorporating the variables of the median of each quartile into the regression model.

To verify the robustness of the results, we further calculated biological aging acceleration using another method, the KDM-AA (Supplementary Tables S1, S2). Higher EO exposure is significantly associated with increased KDM-AA across all models (Model 1: *β* = 0.63, Model 2: *β* = 0.51, Model 3: *β* = 0.66, all *p*-values <0.001). Quintile analyses indicate significantly higher KDM-AA in the highest exposure groups, with significant trends across all models (*p* for trend <0.001). Logistic regression reveals similar associations, where higher EO exposure is linked to increased odds of KDM-AA in both continuous and quintile analyses. All these demonstrate the robustness of the association between EO exposure and biological age acceleration, even after adjusting for various covariates. It is important to note that other harmful physical and chemical factors, which could have additive or synergistic effects on biological aging, were not considered in the analysis, potentially affecting the observed associations.

### 3.3 Restricted cubic spline regression analysis

**Figure 2:**
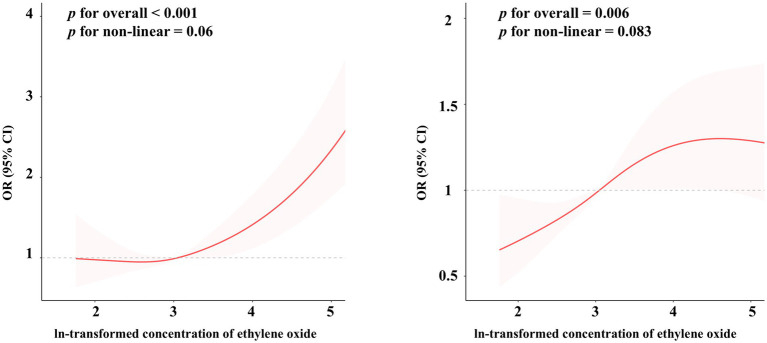
The restricted cubic spline regression between ethylene oxide exposure and the risk of biological age acceleration, weighted. The dose–response relationship was assessed using restricted cubic spline regression, with ethylene oxide exposure (log-transformed) as the predictor, and Phenotypic Age Acceleration **(A)** and Klemera-Doubal Method Age Acceleration **(B)** as the outcomes, adjusted according to Model 3.

The RCS analyses demonstrate significant overall associations between EO exposure and both PhenoAgeAccel (*p* < 0.001) and KDMAC (*p* = 0.006), indicating a meaningful impact of EO exposure on these biological markers (Figure 2). However, the non-linear components for PhenoAgeAccel (*p* = 0.067) and KDMAC (*p* = 0.083) were not statistically significant, suggesting that the associations may be adequately represented by linear relationships rather than complex non-linear trends. These results highlight the significant but potentially linear influence of EO exposure on biological age acceleration and kidney dysfunction markers.

### 3.4 Subgroup analysis and interaction analyses

**Figure 3:**
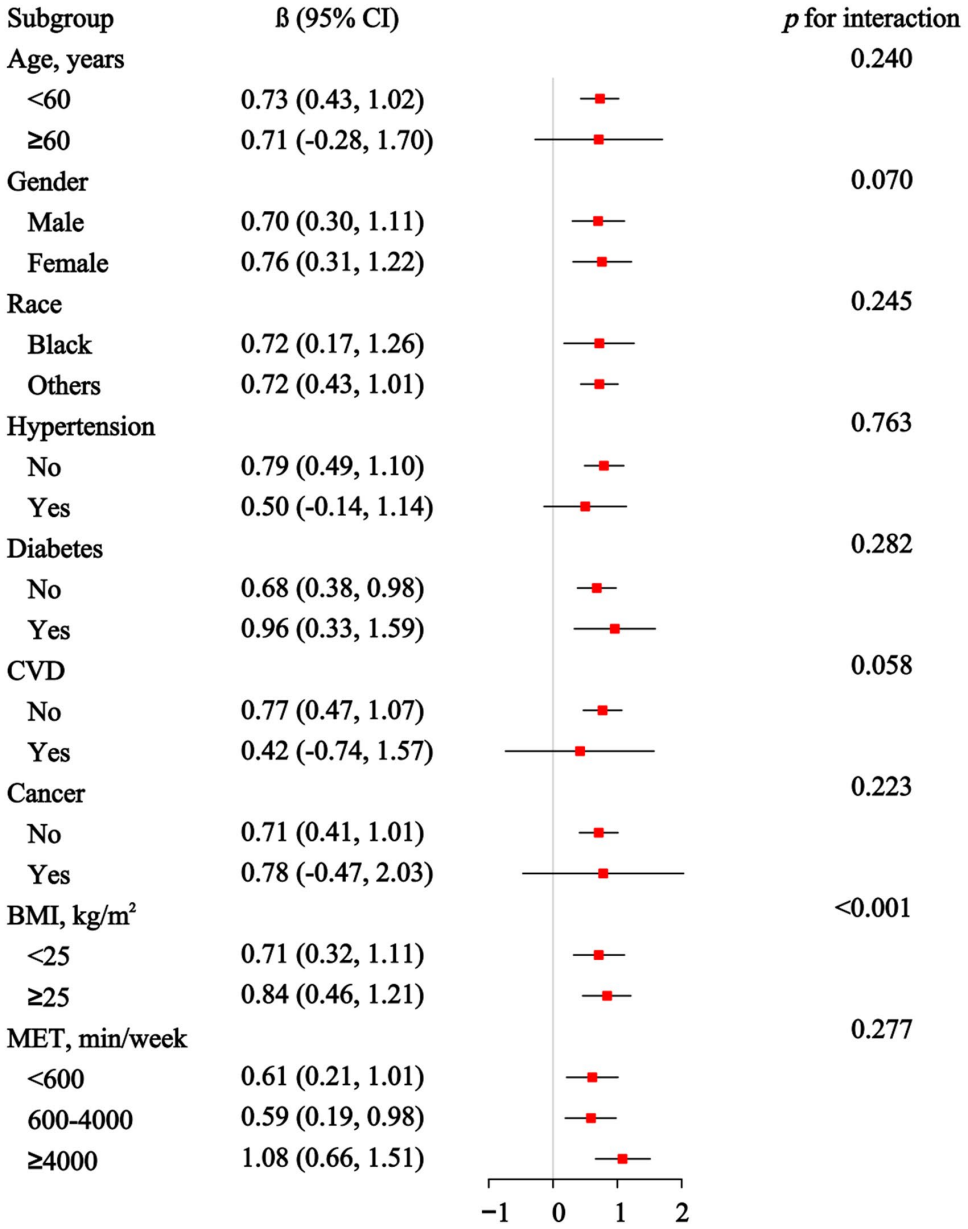
Association between ethylene oxide exposure and Phenotypic Age Acceleration in subgroup and interactive analyses.

The subgroup and interaction analyses presented in Figure 3 indicate the association between EO exposure and PhenoAgeAccel across different demographic and health-related subgroups. However, no significant associations were observed in participants over 60 years old or those with hypertension, diabetes, CVD or cancer. The interaction analyses reveal that BMI has a significant modifying effect (*p* for interaction <0.001). Supplementary Figure S1 shows the subgroup and interaction analyses between EO exposure and KDM-AA. Significant associations are observed in several subgroups, including those under 60 years, non-Hispanic Blacks, participants without hypertension, diabetes or CVD, those with a BMI of 25 or greater, and those with 600–4,000 min of physical activity per week. Interaction analyses reveal significant modifying effects for race, BMI, and physical activity levels.

## 4 Discussion

Our study reveals significant positive associations between EO exposure and accelerated biological aging, even after adjusting for numerous covariates. This suggests that increased EO exposure correlates with heightened biological aging. The consistency of these findings across both PhenoAgeAccel and KDM-AA metrics indicates that EO exposure influences multiple aspects of biological aging. By employing these two distinct metrics, we added an additional layer of validation, demonstrating that the observed associations are not confined to a single method of age estimation. This robustness is further supported by logistic regression analyses, which showed higher odds of accelerated biological aging with increased EO exposure, irrespective of the aging metric employed.

Furthermore, the restricted cubic spline regression analysis in our study indicated a non-linear relationship between EO exposure and PhenoAgeAccel, with a marginally significant non-linear component (*p* = 0.067). This non-linearity suggests a potential threshold effect, where EO exposure may accelerate biological aging particularly at higher levels of exposure. Similar non-linear relationships have been documented in the literature for EO exposure and other diseases. For example, Jiang et al. reported a non-linear dose–response association between EO exposure and epigenetic age acceleration, with more pronounced effects at higher pollutant concentrations (41). This observation is consistent with our findings, indicating that EO exposure may disproportionately intensify its biological impact at elevated levels. In terms of subgroup differences, our study identified significant modifying effects among participants over 60 years old and those with hypertension, cardiovascular disease, or cancer, underscoring the need to consider individual variability in susceptibility to EO exposure. The effect of metabolic health factors, such as diabetes, is closely linked to biological aging, which may be explained by increased vulnerability to oxidative stress and impaired detoxification mechanisms.

The findings of this study align with previous research indicating that environmental pollutants contribute to biological aging. For instance, significant associations have been observed between air pollution exposure and biological aging, as measured by DNA methylation markers (42). Similarly, exposure to polycyclic aromatic hydrocarbons (PAHs) has been linked to reductions in biological age indicators, such as leukocyte telomere length and mitochondrial DNA copy number (43). Additionally, metals like strontium, molybdenum, copper, rubidium, and cobalt have been associated with accelerated aging (44). These findings emphasize the impact of environmental pollutants on biological aging and highlight the importance of preventing exposure to these harmful substances.

The biological mechanisms linking high EO exposure levels to accelerated biological aging are currently unknown. One key aspect of EO’s harmful effects involves its interaction with nucleophilic sites in nucleic acids and proteins (45). HbEO, being electrophilic, reacts with these nucleophilic sites. This covalent binding with nucleophilic groups in DNA can occur after exposure to chemicals or metabolites like EO, acrylamide, and glycidamide, leading to genetic mutations and cancer (10, 45). EO’s role as an alkylating agent capable of inducing DNA damage and mutations may contribute significantly to its impact on biological aging. Additionally, long-term chronic exposure to EO can also lead to reduced glutathione reductase activity and increased hepatic lipid peroxidation, which are associated with oxidative stress *in vivo* (46, 47). Oxidative stress is a well-documented pathway contributing to aging and age-related diseases. The imbalance between the production of ROS and the body’s ability to detoxify these reactive intermediates or repair the resulting damage can accelerate cellular aging processes. Reduced glutathione reductase activity, observed with EO exposure, compromises the cellular antioxidant defense system, thereby increasing vulnerability to oxidative damage. Exposure to EO can also cause an inflammatory response in rodent organs (48). Additionally, the inflammatory response induced by EO exposure could further contribute to aging acceleration. Chronic inflammation is recognized as a hallmark of aging, often referred to as “inflammaging.” Inflammatory cytokines and other mediators can promote cellular senescence, tissue degradation, and various age-related pathologies. Studies have shown that EO exposure can upregulate pro-inflammatory markers, suggesting that inflammation might be a critical mediator in EO-induced biological aging.

The strengths of this study are underscored by the use of a large, nationally representative NHANES dataset, which enhances both the statistical power and generalizability of the findings. Incorporating multiple biological age acceleration metrics, namely PhenoAgeAccel and KDM-AA, strengthens the study’s validity through cross-validation, thereby improving the reliability and robustness of the observed associations. Additionally, subgroup and interaction analyses reveal factors that may influence the relationship between EO exposure and age acceleration, highlighting significant associations within specific demographics. These findings could help guide the development of targeted interventions.

Several limitations should be acknowledged. The cross-sectional design of NHANES limits the ability of this study to establish causality, meaning that the observed associations between EO exposure and biological age acceleration cannot confirm a causal relationship due to the indeterminate temporal sequence between exposure and outcome. Additionally, the inclusion of numerous covariates introduces the risk of multicollinearity, which constrained the number of factors adjusted for in the analysis. Unmeasured confounders may still influence the association between EO exposure and age acceleration. For instance, exposure to other harmful physical and chemical pollutants with potential additive or synergistic effects on biological aging was not accounted for. Furthermore, geographical location, which affects EO exposure through factors such as proximity to industrial facilities, regulatory enforcement, and access to healthcare, was not adjusted for, as NHANES does not provide detailed geographical data. The study also lacks a proposed mechanism for EO’s impact on biological aging. Although existing literature suggests EO’s role in generating reactive oxygen species and its potential genotoxic effects, these mechanisms were not directly measured. Future research should confirm these findings using prospective study designs. Additionally, mechanistic studies are needed to elucidate the underlying biological pathways by which EO exerts its effects on aging. Understanding these pathways could pave the way for targeted interventions to reduce the health burden of EO exposure. Moreover, comparing EO’s effects with those of other environmental pollutants could help contextualize its impact within the broader spectrum of environmental health risks and inform policy decisions aimed at minimizing exposure in both occupational and community settings.

## 5 Conclusion

A significant positive association between EO exposure and biological age acceleration, as measured by both PhenoAgeAccel and KDM-AA after adjusting for various covariates. The RCS analysis suggests a significant, likely linear, association between EO exposure and biological age acceleration. Interaction analyses show that factors such as BMI, race, and physical activity significantly modify the association. These findings collectively underscore the role of EO as a significant factor in biological aging, although other unmeasured factors could contribute to the observed effects.

## Data Availability

The datasets presented in this study can be found in online repositories. The names of the repository/repositories and accession number(s) can be found at: the datasets generated and analyzed during the current study are publicly available from the National Center for Health Statistics at https://wwwn.cdc.gov/nchs/nhanes.

## Footnotes

^1^https://www.who.int/news-room/fact-sheets/detail/ageing-and-health

^2^https://www.cdc.gov/nchs/nhanes/

^3^http://github.com/dayoonkwon/BioAge

^4^https://www.R-project.org
