# Supplementary material for: Associations between ethylene oxide exposure and biological age acceleration: evidence from NHANES 2013–2016
Source: Front Public Health. 2024 Nov 27;12:1488558. doi: 10.3389/fpubh.2024.1488558 (PMC11631939; doi:10.3389/fpubh.2024.1488558)
Supplement: Supplementary file 1 [file Data_Sheet_1.docx]

**Supplemental files**

**Table S1 Linear regression analysis for the associations between ethylene oxide exposure and Klemera-Doubal Method Age Acceleration, weighted**

|  | **Klemera-Doubal Method** **Age Acceleration (KDM-AA)** | | | | | |
| --- | --- | --- | --- | --- | --- | --- |
|  | **Model 1** | | **Model 2** | | **Model 3** | |
|  | **β (95%CI)** | ***p-*value** | **β (95%CI)** | ***p-*value** | **β (95%CI)** | ***p-*value** |
| Continuous^†^ | 0.63 (0.38, 0.88) | <0.001 | 0.51 (0.26, 0.76) | <0.001 | 0.64 (0.27, 1.02) | <0.001 |
| Categories | | | | | | |
| Quintiles 1 | Reference | | Reference | | Reference | |
| Quintiles 2 | 0.08 (-0.62, 0.78) | 0.822 | 0.23 (-0.46, 0.92) | 0.521 | 0.003 (-0.67, 0.68) | 0.993 |
| Quintiles 3 | 1.47 (0.73, 2.21) | <0.001 | 1.66 (0.92, 2.40) | <0.001 | 1.40 (0.67, 2.12) | <0.001 |
| Quintiles 4 | 1.73 (1.00, 2.46) | <0.001 | 1.43 (0.71, 2.16) | <0.001 | 1.44 (0.42, 2.46) | 0.006 |
| *p* for trend |  | <0.001 |  | <0.001 |  | <0.001 |

Model 1 was crude model. Model 2 was adjusted for age + sex + race. Model 3 was adjusted for Model 2 +education level + alcohol consumption + smoking status + physical activity + CVD + hypertension + diabetes + cancer. Continuous†, ln-transformed concentration of ethylene oxide. *p* for trend was tested by incorporating the variables of the median of each quartile into the regression model.

**Table S2 logistic regression analysis for the associations between ethylene oxide exposure and the risk of and Klemera-Doubal Method Age Acceleration, weighted**

|  | **Klemera-Doubal Method Age Acceleration (KDM-AA)** | | | | | |
| --- | --- | --- | --- | --- | --- | --- |
|  | **Model 1** | | **Model 2** | | **Model 3** | |
|  | **OR (95%CI)** | ***p-*value** | **OR (95%CI)** | ***p-*value** | **OR (95%CI)** | ***p-*value** |
| Continuous^†^ | 1.12 (1.05, 1.21) | 0.002 | 1.10 (1.03, 1.19) | 0.011 | 1.18 (1.03, 1.36) | 0.021 |
| Categories | | | | | | |
| Quintiles 1 | Reference | | Reference | | Reference | |
| Quintiles 2 | 1.18 (0.90, 1.56) | 0.215 | 1.22 (0.92, 1.61) | 0.152 | 1.19 (0.90, 1.57) | 0.207 |
| Quintiles 3 | 1.43 (0.98, 2.08) | 0.059 | 1.49 (1.02, 2.19) | 0.042 | 1.46 (0.99, 2.15) | 0.057 |
| Quintiles 4 | 1.45 (1.13, 1.85) | 0.004 | 1.39 (1.07, 1.80) | 0.015 | 1.51 (1.06, 2.16) | 0.027 |
| *p* for trend |  | 0.003 |  | 0.008 |  | 0.008 |

OR: weighted odds ratio. Model 1 was crude model. Model 2 was adjusted for age + sex + race. Model 3 was adjusted for Model 2 +education level + alcohol consumption + smoking status + physical activity + CVD + hypertension + diabetes + cancer. Continuous†, ln-transformed concentration of ethylene oxide. *p* for trend was tested by incorporating the variables of the median of each quartile into the regression model.


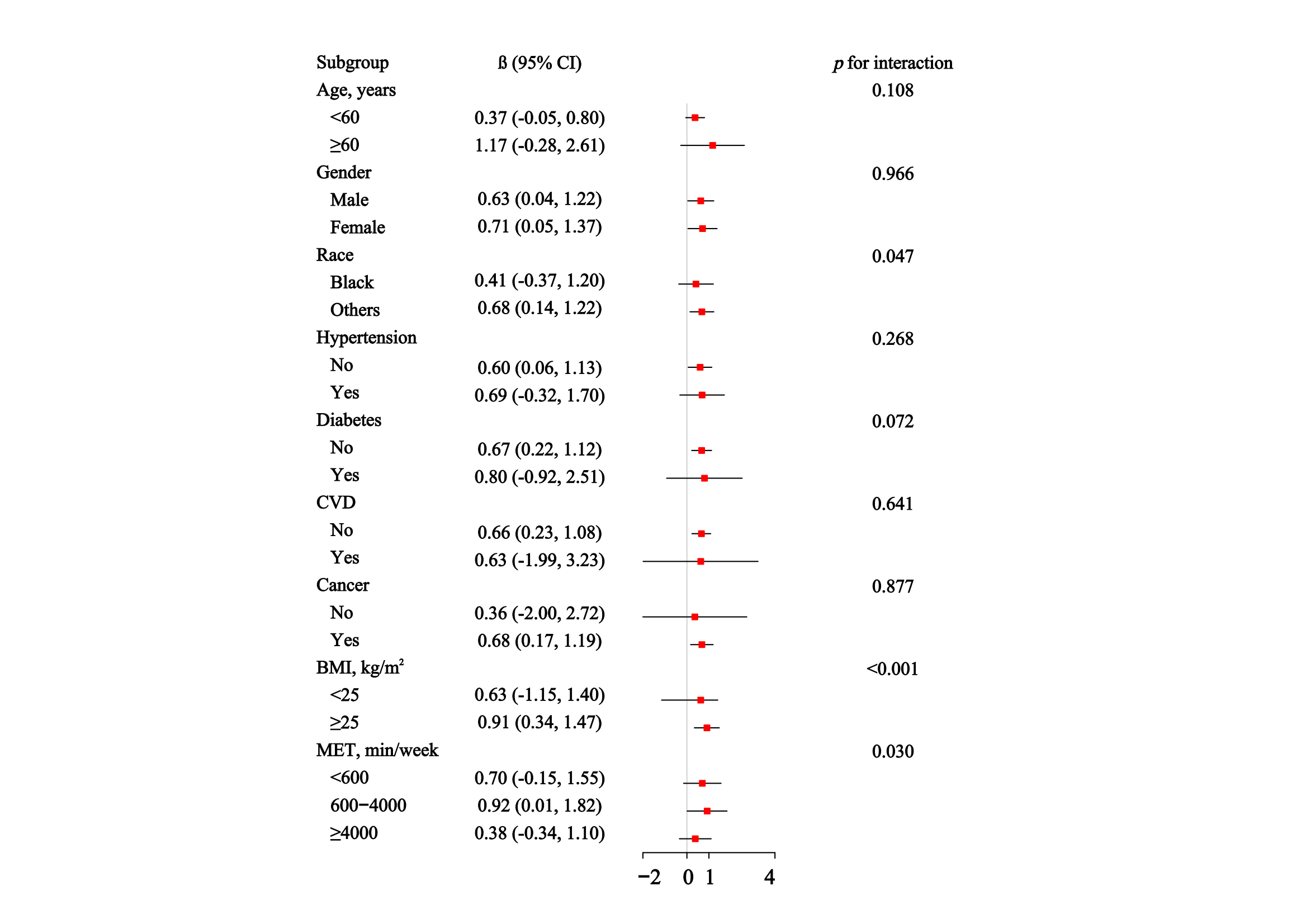


**Fig. S1 Association between ethylene oxide exposure and KDM-AA in subgroup and interactive analyses.**
